# Sinus Valsalva Aneurysm of the non-coronary cusp initially diagnosed as right ventricular thrombus: A case report

**DOI:** 10.1016/j.radcr.2021.10.063

**Published:** 2021-11-25

**Authors:** Sulayman el Mathari, Teun van der Bom, Berto Bouma, Antoine Driessen, Jolanda Kluin

**Affiliations:** a Department of Cardiothoracic Surgery, Amsterdam University Medical Center, Meibergdreef 9, 1105 AZ, Amsterdam, Netherlands; b Department of Cardiology, Amsterdam University Medical Center, Meibergdreef 9, 1105 AZ, Amsterdam, Netherlands

**Keywords:** Sinus of Valsalva aneurysm, Right ventricular thrombus, Cardiac surgery, ECG-gated computed tomography, Transthoracic echocardiography

## Abstract

Sinus of Valsalva aneurysms are abnormal bulges of the aortic root caused by a tissue deficiency resulting in an enlargement of the aortic root in the area between the aortic annulus and the sinotubular junction. Frequently, sinus of Valsalva aneurysms are asymptomatic. However, sinus of Valsalva aneurysms can also be potentially fatal due to their risk of rupture. We present a case of a 49-year old asymptomatic male patient with a rare image of a sinus of Valsalva aneurysm of the non-coronary cusp which was initially mistaken for a right ventricular thrombus. Surgical repair of the sinus of Valsalva aneurysm was eventually achieved by a valve sparing root replacement and the patient was discharged 7 days after surgery. This case report shows that sinus of Valsalva aneurysms are vulnerable to misdiagnosis when it is not initially suspected. Because of the potential fatality of this phenomenon we would like to underline the necessary vigilance in the diagnostic process, as sinus of Valsalva aneurysms can be missed when the physician is not aware of this potential rare diagnosis.

## Background

Sinus of Valsalva aneurysms (SVAs) are abnormal bulges of the aortic root in the area between the aortic annulus and the sinotubular junction. This phenomenon is caused by a tissue deficiency in the elastic lamina leading to degeneration of the aortic media layer [1]. Its estimated rate of incidence in the general population is 0.09% [2]. In most cases, SVAs are asymptomatic. Therefore, they are rarely observed unruptured and often only come to light when a patient becomes symptomatic due to rupture. This is potentially fatal because of its risk for rapid hemodynamic deterioration due to aorto-cardiac shunting. In such cases, immediate surgical treatment is required [3]. SVAs which are discovered before rupture can be repaired surgical in an elective setting. This can be achieved either with a valve-sparing aortic root replacement or a combined root and valve replacement, also known as the Bentall procedure [4,5]. The latter depends on the involvement and pathology of the aortic valve. In this paper we describe a case report of a 49-year old man with an asymptomatic SVA of the non-coronary cusp (NCC) which was initially diagnosed as a right ventricular thrombus.

## Case report

**Fig. 1:**
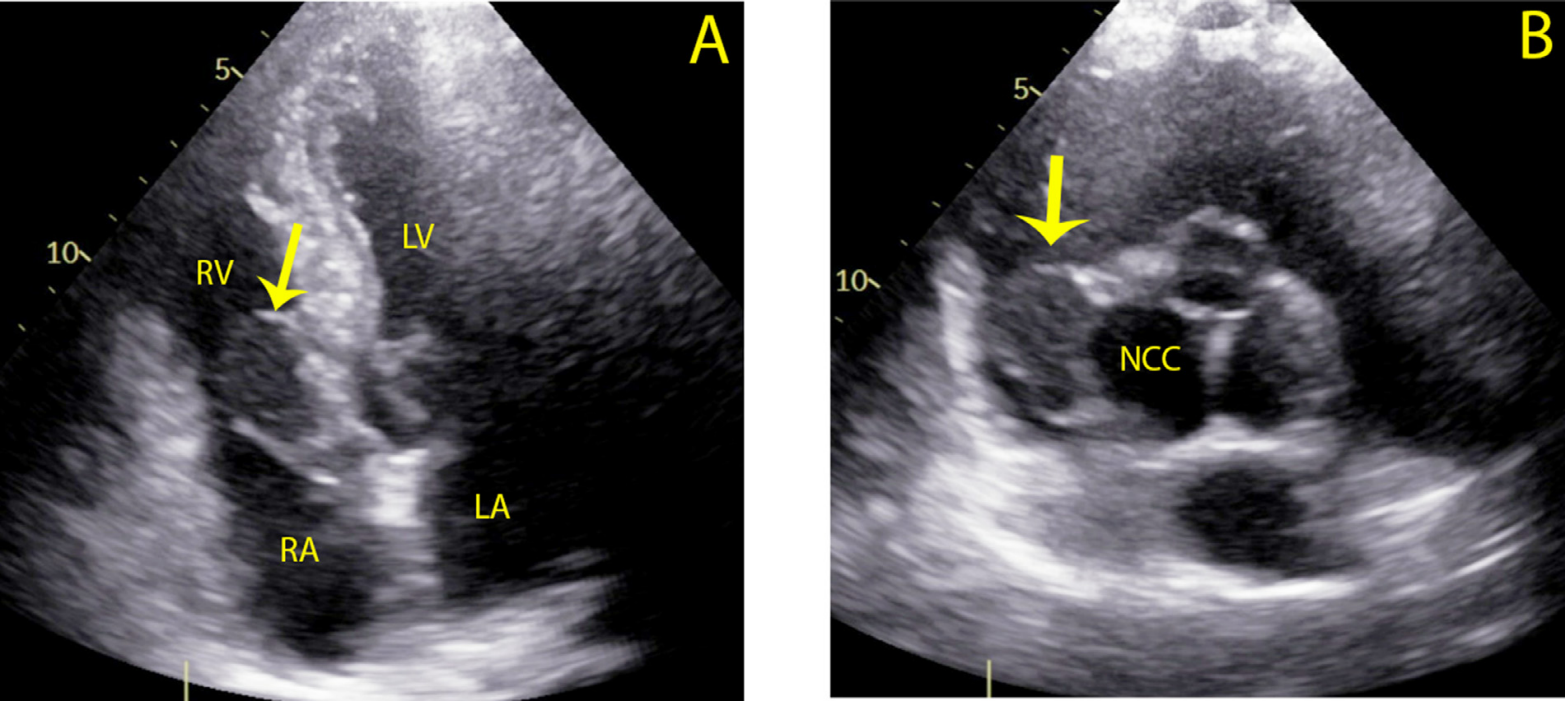
Transthoracic Echocardiography with image of hypo-echogenic structure (yellow arrow) in the right ventricular **(RV)** cavity in apical four-chamber view (panel A) and parasternal short-axis view (panel B). *LV: left ventricle, LA: left atrium, NCC: non-coronary cusp, RV: right ventricle, RA: right atrium.*

**Fig. 2:**
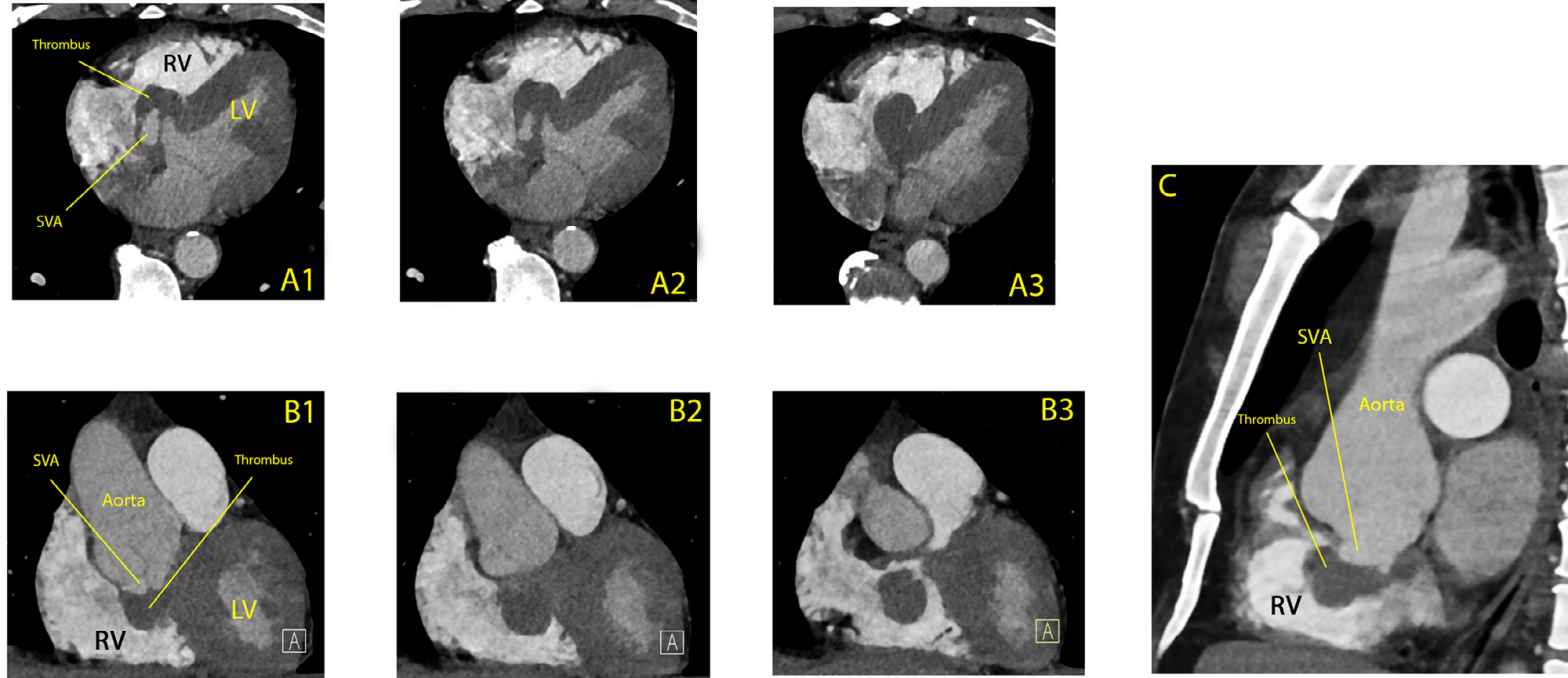
Cardiac Computed Tomography Angiography scan with images of a Sinus Valsalva Aneurysm (SVA) of the non-coronary cusp bulging into the right ventricle. Images are shown in axial (panel A1-A3), coronal (panel B1-B3) and sagittal (C) view. *RV: right ventricle, LV: left ventricle.*

A 49-year old asymptomatic male was admitted to our institution after referral from his cardiologist in an outpatient care clinic, where he was monitored because of a dilated aortic root (46 mm). This was an incidental finding by transthoracic echocardiography (TTE) 6 months earlier at presentation due to non-specific chest pain. Except for hypertension, the patient had no other history of disease. Now 6 months later, the most recent TTE did not only show an increase in the aortic root diameter (50 mm), but also a hypo-echogenic structure in the right ventricular cavity **(**Fig. 1**)**. The latter was interpreted as a right ventricular thrombus, position-wise possibly related to the tricuspid valve. Moreover, because of the rapid increase of the aortic diameter (4 mm in 6 months), the patient was referred to our hospital where a regular non-gated computed tomography angiography (CTA) scan was performed for acute aortic pathology. At the initial evaluation of the scan there was no suspicion of acute aortic pathology besides the dilation. After this CTA, the patient was discharged home with the intention to perform magnetic resonance imaging (MRI) a week later to determine the nature of the right ventricular structure. However, upon review of the CTA the next morning there was a suspicion of aortic dissection, based upon a possible tear in the aortic root. The patient was readmitted urgently for a new and dedicated electrocardiogram-gated (ECG-gated) cardiac computed tomography angiography (CCTA) scan **(**Fig. 2**)** on which a structure of hypodense tissue was observed to derive from the sinus of Valsalva of the NCC bulging into the right ventricular cavity with involvement of the perimembranous septum. This suggested it to be a SVA filled with thrombus. As there was no continuation of contrast from the aortic root to the right ventricular cavity, a rupture was highly unlikely. However, given the potential risk for rupture with the rapid increase in aortic diameter and the thrombus suggesting the possibility of bleeding, the patient was referred for urgent cardiac surgery.

**Fig. 3:**
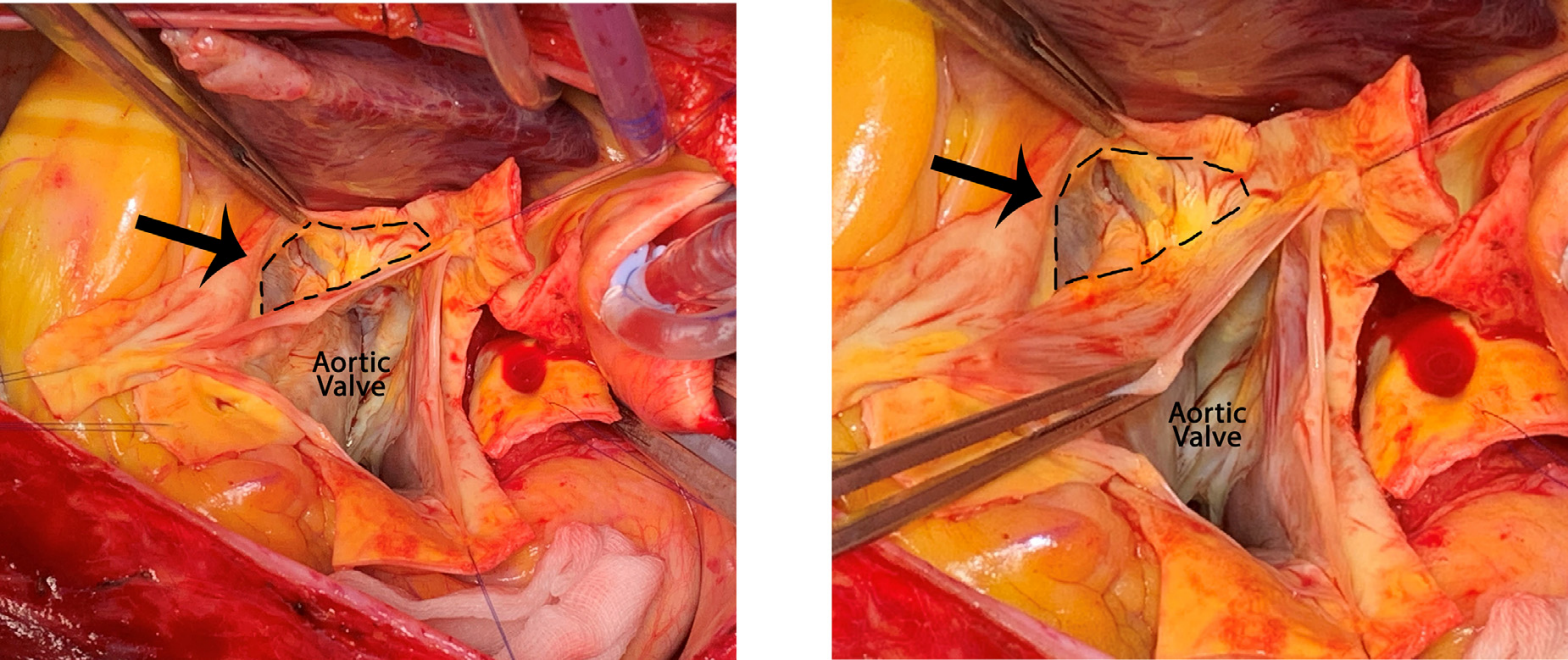
Perioperative aortic root with a tricuspid aortic valve. The arrow is pointing at the sinus of Valsalva aneurysm of the non-coronary cusp which is bulging into the right ventricular cavity. There was no thrombus present.

During surgery, a SVA of the NCC bulging to the right ventricular cavity was observed **(**Fig. 3**)**. There was no thrombus inside the aneurysm. As the native aortic valve was tricuspid and functioned well, a valve-sparing root replacement was performed. Histopathological analysis of the excised aortic tissue showed fragmentation of the elastic lamina in line with aortic media degeneration and no signs of connective tissue disease. This fits the general known cause of SVAs. There were no relevant complications in the postoperative setting and the patient was discharged 7 days after surgery.

## Discussion

SVAs are rarely diagnosed in asymptomatic patients due to their low incidence (0.09%) and the relatively low specificity on echocardiography. In this case, the SVA on TTE was initially mistaken as a right ventricular thrombus, position-wise related to the tricuspid valve. A regular non-gated CTA was performed to exclude aortic pathology. However, the SVA was missed. Only after revision, the aortic root on the primary CTA were deemed to be abnormal. With a redo dedicated ECG-gated CCTA the next day, the diagnosis of SVA was established.

Early surgical correction of the SVA in this patient was chosen because of the risk of rupture due to the rapid expansion of the aortic diameter with 4 mm in 6 months and the observed possible thrombus on imaging suggesting a possible bleeding. This is in line with the recommendation for SVA treatment to prevent a rupture resulting in a critical hemodynamic pathophysiology, also in case of asymptomatic patients [6].

So far, there is only one case report known in literature of a patient with a comparable preoperative image [7]. In this case there was a SVA of the left coronary cusp bulging into the left atrium. Here, perioperative assessment showed an aneurysm thrombus in place of the hypodense area on imaging. Yet, despite the preoperative imaging similarities with this case, we did not find an aneurysm thrombus during surgery. This is probably due to the RV not being assessed intraoperative because there was no need to. In retrospect our hypothesis is that this hypodense area is a subendocardial hemorrhage in the RV derived from the SVA of the NCC.

In conclusion, we would like to underline the importance of appropriate usage of imaging in SVAs and the necessary vigilance for this potential diagnosis. This case shows that SVAs are vulnerable to misdiagnosis, when it is not initially suspected. TTE is known in literature for having a high sensitivity in diagnosing SVAs and thus being the gold standard [3]. However, as shown in this case, we would recommend extensive assessment using dedicated ECG-gated CCTA imaging for complete determination as this seems to have a better diagnostic specificity.

## Patient consent

The authors obtained written informed consent from the patient for submission of this manuscript for publication.
